# Understanding Mechanisms Underlying Non-Alcoholic Fatty Liver Disease (NAFLD) in Mental Illness: Risperidone and Olanzapine Alter the Hepatic Proteomic Signature in Mice

**DOI:** 10.3390/ijms21249362

**Published:** 2020-12-08

**Authors:** Bahman Rostama, Megan Beauchemin, Celeste Bouchard, Elizabeth Bernier, Calvin P. H. Vary, Meghan May, Karen L. Houseknecht

**Affiliations:** 1 Department of Biomedical Sciences, College of Osteopathic Medicine, University of New England, Biddeford, ME 04005, USA; brostama@une.edu (B.R.); mbeauchemin1@une.edu (M.B.); cbouchard2@une.edu (C.B.); mmay3@une.edu (M.M.); 2 Department of Psychology, University of Southern Maine, Portland, ME 04103, USA; elizabeth.bernier@maine.edu; 3 Center for Molecular Medicine, Maine Medical Center Research Institute, Scarborough, ME 04074, USA; VARYC@MMC.ORG

**Keywords:** antipsychotic, liver, proteome, energy metabolism, nonalcoholic fatty liver disease, steatosis, schizophrenia, inflammation, autonomic nervous system

## Abstract

Patients with severe mental illness have increased mortality, often linked to cardio-metabolic disease. Non-alcoholic fatty liver disease (NAFLD) incidence is higher in patients with schizophrenia and is exacerbated with antipsychotic treatment. NAFLD is associated with obesity and insulin resistance, both of which are induced by several antipsychotic medications. NAFLD is considered an independent risk factor for cardiovascular disease, the leading cause of death for patients with severe mental illness. Although the clinical literature clearly defines increased risk of NAFLD with antipsychotic therapy, the underlying mechanisms are not understood. Given the complexity of the disorder as well as the complex pharmacology associated with atypical antipsychotic (AA) medications, we chose to use a proteomic approach in healthy mice treated with a low dose of risperidone (RIS) or olanzapine (OLAN) for 28 days to determine effects on development of NAFLD and to identify pathways impacted by AA medications, while removing confounding intrinsic effects of mental illness. Both AA drugs caused development of steatosis in comparison with vehicle controls (*p* < 0.01) and affected multiple pathways relating to energy metabolism, NAFLD, and immune function. AA-associated alteration in autonomic function appears to be a unifying theme in the regulation of hepatic pathology.

## 1. Introduction

Patients with severe mental illness, including schizophrenia, experience a significantly shortened life expectancy with high rates of all-cause mortality [1,2,3], most commonly associated with metabolic dysregulation and cardiovascular disease. Non-alcoholic fatty liver disease (NAFLD) is a growing epidemic worldwide and is associated with obesity, insulin resistance, and inflammation [4,5,6,7]. Incidence of NAFLD is elevated in patients with severe mental illness [3] and with atypical antipsychotic (AA) treatment [8]. NAFLD is progressive in nature, characterized by hepatic steatosis and fibrosis, and can culminate in cirrhosis and hepatocarcinoma if not managed [9,10,11]. NAFLD is the most prevalent chronic liver disease in the world, and given the exponential rise in incidence of NAFLD [12], this disease is now one of the leading indications for hepatic transplant [13]. Understanding drug-associated effects on NAFLD incidence and progression is therefore of growing importance.

Among patients with severe mental illness, metabolic dysregulation can be due to intrinsic factors as well as extrinsic factors, including antipsychotic medications [14]. Although the clinical literature describes relative risks and contributions of intrinsic and extrinsic effects on metabolic disorders in schizophrenia [15,16,17], underlying mechanisms in both schizophrenic and non-schizophrenic patients remain elusive. Patients prescribed antipsychotic medications experience significant endocrine and metabolic side effects including weight gain, insulin resistance, hyperglycemia, dyslipidemia, hyperprolactinemia, and bone loss, regardless of diagnosis [6,18]. Broadly stated, AA side effects appear to be mediated both by global (i.e., the central nervous system) and tissue-specific mechanisms, including those in the liver.

Multiple groups have explored underlying mechanisms using preclinical models in order to avoid confounding intrinsic risks present in patient populations. Given the tremendous complexity of the known AA pharmacology as potent antagonists at multiple G-protein coupled receptors (GCPR), as well as the complex endocrine, metabolic, and immune side effects observed clinically and pre-clinically across the lifespan, we chose to take a proteomic approach to evaluate the effects of AA medications on protein expression profiles in liver. The goal of this study was to elucidate AA-regulated pathways associated with the development of NAFLD. To address this aim, we chose two widely prescribed AA drugs with divergent pharmacology [19] and differences in metabolic risk [20]: risperidone (RIS) and olanzapine (OLAN). OLAN is associated with high clinical risk for obesity, dyslipidemia, and other aspects of insulin resistance/metabolic syndrome, while RIS is known to have intermediate metabolic liability.

We provide the first report (to our knowledge) of hepatic proteomic signatures following sub-chronic treatment with clinically relevant doses of RIS and OLAN, and report their diversity from the hepatic proteome of drug vehicle (VEH)-treated controls using healthy adult mice. These data identify canonical pathways impacted with AA treatment that are linked to metabolic and immunologic dysregulation and are consistent with drug-associated increase in the incidence of NAFLD and enhanced sympathetic tone.

## 2. Results

### 2.1. Animal Health

All animals used in this study were healthy and displayed no adverse effects of daily gavage for the 28-day study duration. Details of animal growth rates, locomotor activity, adiposity, and energy expenditure for this cohort were recently reported in a manuscript describing AA effects on the heart [21]. Overall, the animals in this study showed normal feeding behavior, weight gain, and locomotor activity. Although there was no treatment effect on body weight gain, animals treated with RIS or OLAN showed a significant increase in adiposity compared to VEH treatment as well as a reduction in plasma-free fatty acid concentrations [21]. Importantly, animals were maintained on standard mouse chow to avoid potentially confounding effects of high-fat feeding, an experimental paradigm associated with diet-induced obesity and NAFLD [22,23]. Consistent with this dietary regimen, there was no treatment effect on concentrations of total plasma triglycerides (299 µM ± 55.1, 291 µM ± 40.0, 247 µM ± 21.6; *p* > 0.05) or total cholesterol (200 µM ± 13.4, 205 µM ±7.93, 213 µM ± 10.7; *p* > 0.05) in VEH, RIS, or OLAN mice, respectively.

### 2.2. Atypical Antipsychotic Treatment Induced Histopathologic Changes Consistent with the Development of NAFLD

**Figure 1:**
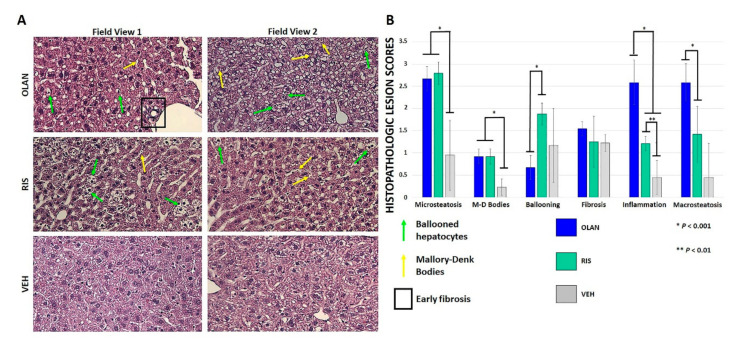
Atypical antipsychotic (AA) treatment led to histopathologic changes consistent with early non-alcoholic fatty liver disease (NAFLD). Hematoxylin and eosin staining showed several histopathologic changes at 20× magnification following AA treatment relative to vehicle (VEH)-treated controls (**A**). These included micro- and macrosteatosis, ballooned hepatocytes (green arrows), Mallory–Denk bodies (yellow arrows), and small areas of early fibrosis (boxed). Two representative field views are shown. Statistical significance (*, **) in lesion scores between AA-treated mice and VEH-treated mice was found for microsteatosis, Mallory–Denk body formation, and formation of inflammatory foci (**B**).

Moderate duration AA treatment induced microsteatosis, macrosteatosis, ballooning hepatocytes, inflammatory foci, and Mallory–Denk body formation in healthy mice, as shown across two representative field views in Figure 1A. Significant differences in histopathologic lesion scores between RIS or OLAN and VEH controls were found for microsteatosis, Mallory–Denk body formation, and formation of inflammatory foci (Figure 1B). Significant differences in hepatocyte ballooning and macrosteatosis were found for RIS and OLAN, respectively. There was early evidence of periportal fibrosis occurring sporadically in OLAN-treated mice, although this did not rise to statistical significance. Lesions generated by RIS and OLAN were largely consistent with early NAFLD.

### 2.3. Atypical Antipsychotic Treatment Altered the Hepatic Proteome

We evaluated the effect of sub-chronic (28 day) AA treatment on hepatic protein expression using an unbiased sequential window acquisition of all theoretical spectra (SWATH) proteomic approach to characterize the unique protein signature resulting from RIS or OLAN treatment, compared to VEH (see Supplementary File S1). The proteomic signature data were then curated by analyzing the Kyoto Encyclopedia of Genes and Genomes (KEGG) database pathway maps for the role of each protein and the resulting changes in predicted physiological functions, which were cataloged and sorted for up- or downregulation relative to vehicle controls (see Supplementary Files S2–S4). Importantly, the proteomic analysis was conducted using liver samples from the same animals that we evaluated histologically (Figure 1), and thus proteomic results are coincident with AA-associated NAFLD.

**Figure 2:**
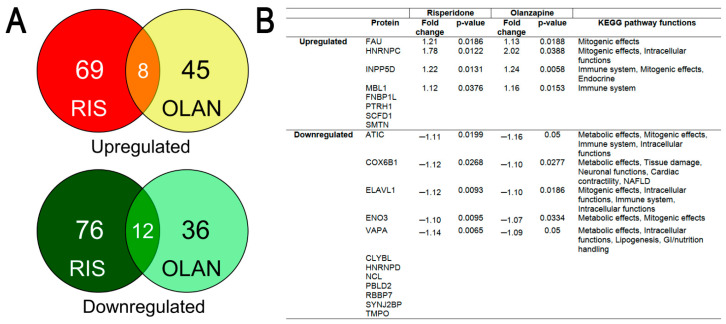
Proteomic analysis of hepatic proteins differentially expressed (DE) by both risperidone and olanzapine. (**A**) Venn diagrams showing shared proteins that are significantly upregulated (top) or downregulated (bottom) in risperidone (RIS)- or olanzapine (OLAN)-treated mice (*p* < 0.05). (**B**) Of the 20 shared proteins, 9 were found to have KEGG (Kyoto Encyclopedia of Genes and Genomes) pathway maps from which predicted functional changes could be determined. Statistical significance (*p* < 0.5) of differentially expressed (DE) proteins relative to vehicle (VEH). RIS *n* = 5; OLAN *n* = 4; VEH *n* = 4. Abbreviations: NAFLD, non-alcoholic fatty liver disease; GI, gastrointestinal.

AA treatment was associated with a large number of proteins that were differentially expressed (DE) in the livers of AA-treated mice relative to VEH control (Figure 2A). Livers from RIS mice showed the greatest overall number of proteins changed, with 69 proteins upregulated and 76 proteins downregulated vs. VEH (*p* < 0.05). For OLAN mice, expression of 45 proteins were upregulated, while 46 proteins were downregulated (*p* < 0.05). Further analysis revealed that 20 were similarly changed in RIS and OLAN mice (up- or downregulated; Figure 2A), while no proteins were differentially changed (increased by one drug but decreased by the other).

The DE proteins common between the two treatments were predicted to affect mostly metabolic, mitogenic (growth signaling), and immune functions, on the basis of KEGG pathway analysis (Figure 2B). The highest cumulative number of predicted functional changes caused by both RIS- and OLAN-induced protein dysregulation were metabolic functions, especially disruptions in the ATP production pathways of glycolysis and mitochondrial oxidative phosphorylation. Similarly, many of the same disrupted ATP-generating pathways also predicted decreased amino and nucleic acid synthesis as a result of the dysregulated proteins. Interestingly, some predicted pathway functions related to mitogenic signaling, such as survival (resistance to apoptosis), were increased, and with them the predicted functions that indicated increased probabilities of various cancers, according to the KEGG pathway maps. Additionally common between the two AA treatments were predicted increases in generation of reactive oxygen species and oxidative stress, resulting from the protein changes (Table 1 and Table 2).

Setting a cutoff of 10 combined changes (“up” + “down”) for each KEGG pathway-predicted function, we found that OLAN caused 24 and RIS caused 17 functional pathway changes (Table 1 and Table 2). The complete list can be found in Supplementary File S4. Predicted functional pathway changes with OLAN treatment also fell into a larger number of categories than RIS, including immune effects, gastrointestinal (GI) functions, intracellular functions, and effects on regulation of blood pressure.

Many of the predicted functional changes resulting from the AA treatment-induced protein dysregulation were differentially altered (increased by one drug but decreased by the other), likely due to differences in pharmacological properties of the two medications [19,24]. For example, lipolysis pathway function was more frequently downregulated by OLAN, but was upregulated by RIS. Similarly, pathway changes that predicted increased cell migration by RIS were decreased by OLAN (Table 1 and Table 2).

As with the 20 DE proteins common to RIS and OLAN (Figure 2 panel B), the predicted functions of the other 216 dysregulated proteins also predominantly affected metabolic functions, with predicted reductions in ATP-generating pathways (glycolysis, mitochondrial oxidative phosphorylation, and generation of pyruvate). Nucleic acid synthesis and amino acid synthesis were also predicted to be reduced more frequently, as a result of the up- or downregulation of the proteins. Both RIS and OLAN showed an increase in predicted pathways that led to lipogenesis, which is in line with the observed increases in liver steatosis (Figure 1).

We found that there were some differentially expressed proteins (*p* < 0.05) that were represented in a large and diverse number of KEGG pathway maps (Table 3). Using cutoff criterion of a minimum of five representative KEGG pathways, we found that RIS had 25 proteins (16 upregulated, 9 downregulated) and OLAN had 15 proteins (6 upregulated, 9 downregulated) represented in multiple pathways. These impactful DE proteins featured prominently in signal transduction pathways, mitochondrial functions, and glycolysis.

### 2.4. Antipsychotic Treatment Altered Protein Expression in Hepatic Energy Metabolism Pathways

AA medications alter whole-body energy metabolism and insulin sensitivity both clinically and pre-clinically, including liver-specific effects such as AA-associated increases in hepatic glucose production [25,26,27]. In this study, dysregulation of energy metabolism was a prominent predicted outcome of the AA-induced DE in the liver proteome. Utilization of glucose, lipids, and amino acids in various pathways were disturbed, and this contributed to insulin resistance.

Many of the DE proteins were predicted to impact mitochondrial energy metabolism, including oxidative phosphorylation, the tricarboxylic acid cycle, and the electron transport chain. RIS affected multiple proteins within mitochondria, including COX6B1, ACAA2, BCAT2, ALDH7A1, CS, ECI2, MECR, NDUFA8, NDUFB8, and NDUFS1, and OLAN affected COX6B1, CYCS, and AK2. Some of the proteins affected mitochondrial function from outside of the organelle, such as ACLY and MTHFD1 by RIS, and DECR2 and ENO3 by OLAN.

RIS and OLAN treatment caused different patterns of DE protein expression relating to glycolytic pathways, with RIS effects promoting increases in glycolytic flux pathways mainly by shifts away from mitochondrial function and increased pro-growth pathways, but OLAN had reduced glycolytic pathway modulation, mainly due to decreased glycolytic enzymes (Table 3).

The most frequent contributing factor to the predicted overall decrease in energy generation for both RIS and OLAN were the DE proteins predicting reduced generation of pyruvate via metabolism of amino acids and other metabolites. With RIS, this included downregulation of AGXT, ALDH7A1, ENO3, CS, ABAT, TALDO1, and AOX3, and with OLAN the downregulation of ENO3, PNP, AMY2B, VAPA, AK2, and ATP2B4.

Insulin resistance can lead to hyperglycemia, and several of the DE proteins in both AA treatment groups were associated with pathways linked to disrupted circulating glucose homeostasis and insulin resistance. RIS induction of INPP5D was predicted to decrease insulin receptor signaling, and its downregulation of ALR1 was predicted to affect multiple upstream glucose processing pathways. RIS also increased ADIPOQ, whose adipokine functions are predicted to improve insulin sensitivity. OLAN’s downregulation of the two important glycolytic enzymes ENO3 and AK2 were predicted to increase hyperglycemia.

Ectopic lipid accumulation (lipotoxicity) is associated with insulin resistance [28,29,30], and predicted dysregulation of lipid metabolism was evident in both the RIS and OLAN treatment groups. Lipotoxicity was predicted by RIS treatment via changes in processing of fatty acids and coenzyme-A by the DE proteins NUDT7, AOX3, ACAA2, and ECI2. OLAN’s effects on lipotoxicity were predicted by changes to fatty acid oxidation and cholesterol transport by the DE proteins CYCS, COX6B1, DECR2, NPC2, and GNAI1. RIS also had multiple predicted interactions with the peroxisome proliferator-activated receptors (PPARs), which are transcription factors regulating expression of genes involved in various aspects of metabolism including lipid metabolism [31,32]. RIS increased ADIPOQ, which is associated with regulation of adipocyte differentiation, lipid uptake, and insulin sensitivity [33,34,35], as well as SMARCC1, a co-transcription factor of PPARγ. RIS also increased SLC27A1, whose fatty acid transport function is predicted to activate all PPARs (alpha, beta/delta, and gamma). At the RNA level, OLAN treatment significantly altered expression of 26 genes (*p <* 0.05) that are regulated by PPARs (Supplementary File S5). Notably, FABP3, lipoprotein lipase and UCP1 were upregulated > 3-fold vs. VEH.

### 2.5. Antipsychotic Treatment Altered Expression of Proteins Associated with NAFLD

**Figure 3:**
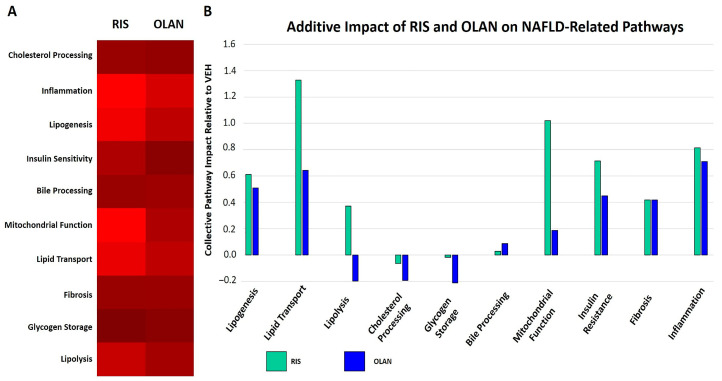
Dysregulation of pathways leading to NAFLD during risperidone and olanzapine treatment. DE proteins measured in this study are integral parts of several pathways in the KEGG database known to contribute to NAFLD. The number of proteins altered in RIS- or OLAN-treated mice (relative to VEH-treated controls) in each pathway group were tabulated (**A**). The intensity of red (λ = 100–255 nm) reflects the number of proteins per pathway significantly different from VEH-treated mice during RIS treatment (left panel) and OLAN treatment (right panel). The aggregate changes in functionality of pathways across proteins compared to VEH-treated mice for RIS and OLAN are depicted (**B**). RIS and OLAN dysregulated inflammation, fibrosis, insulin resistance, and lipogenesis at comparable levels, but vary markedly in their impact on pathways related to lipolysis, lipid transport, glycogen storage, cholesterol processing, and mitochondrial function.

Some of the DE proteins among the RIS and OLAN lists were those with predicted functional effects associated with liver steatosis, fibrosis, and NAFLD (Figure 3). Metabolic syndrome and insulin resistance contribute greatly to the progression of AA-mediated liver disease, including steatosis and fibrosis [8,36]. Among the KEGG pathway-predicted functional changes, metabolism featured prominently for both RIS and OLAN.

Several DE proteins are linked to pathways that regulate fibrosis. In the RIS treatment group, increased PRKCA signaling was predicted to increase activation of the Transforming Growth Factor-beta (TGF-β) pathway, leading to increased matrix deposition, and decreased AOX3 would lead to decreased retinoic acid, both leading to increased fibrosis. In the OLAN treatment group, increased PLOD3 activity was predicted to increasingly hydroxylate and glycosylate collagen motif-containing proteins, leading to cross-linking and fiber formation [37].

The predicted effects of the DE of some proteins also indicated potential insulin resistance, an important cause of NAFLD. With RIS treatment, the decreased expression of AOX3 and increased expression of INPP5D are predicted to contribute to insulin resistance. With OLAN treatment, in addition to its induction of INPP5D, the suppression of AMY2B and GNAI1 are predicted to increase hyperglycemia and decrease insulin sensitivity. Some of the DE proteins predicted to affect mitochondrial function also cause dyslipidemia, including MECR, CS, ACLY, AOX3, and COX6B1 by RIS, and CYCS, COX6B1, and ENO3 by OLAN.

RIS and OLAN were also predicted to impact regeneration and repair pathways, but their functional profiles were different. One important predicted regenerative function was mitochondrial biogenesis, which was essentially absent in the OLAN proteome profile, but promoted by RIS-mediated increase of ADIPOQ, GNAS, PRKCA, PDGFRB, SMARCC1, and SLC27A1.

The suppression of apoptotic pathways led both AAs to have a predicted increase in cancer incidence or severity. RIS had more increases in proteins with pro-growth and pro-mitogenic signaling effects, such that both proliferation and survival were increased. These included increased receptor tyrosine kinases such as EPHA2 and PDGFRB, as well as GNAS (G_s_ G-protein) and PRKCA, which were prominently represented in many KEGG pathways. There were also protein DE that predicted suppression of apoptosis with RIS, such as GSTA3 (increased glutathione activity), and IL1RAP. OLAN, however, saw increases in predicted survival pathways mostly by suppression of apoptosis via decreased expression of CYCS and GNAI1 (G_i_ G-protein), as well as increased G6PD, which contributes to increased glutathione production.

The pathways that indicated increases in cancer incidence and severity (see Table 2 and Table 3) followed the same distinct trends for the two drugs, with RIS promoting growth and survival, and OLAN decreasing apoptotic pathways. However, both RIS and OLAN treatment caused increase in proteins that predicted DNA damage (and impairment in DNA repair). For RIS, these were from the downregulation of MTHFD1 and ATIC, and for OLAN, the downregulation of CTC1 and TOP2B. Some of the predicted functions that indirectly promoted cancer severity included angiogenesis, metastasis, and drug resistance.

### 2.6. Antipsychotic Treatment Disrupted Hepatic Immune/Inflammatory Signaling Pathways

**Figure 4:**
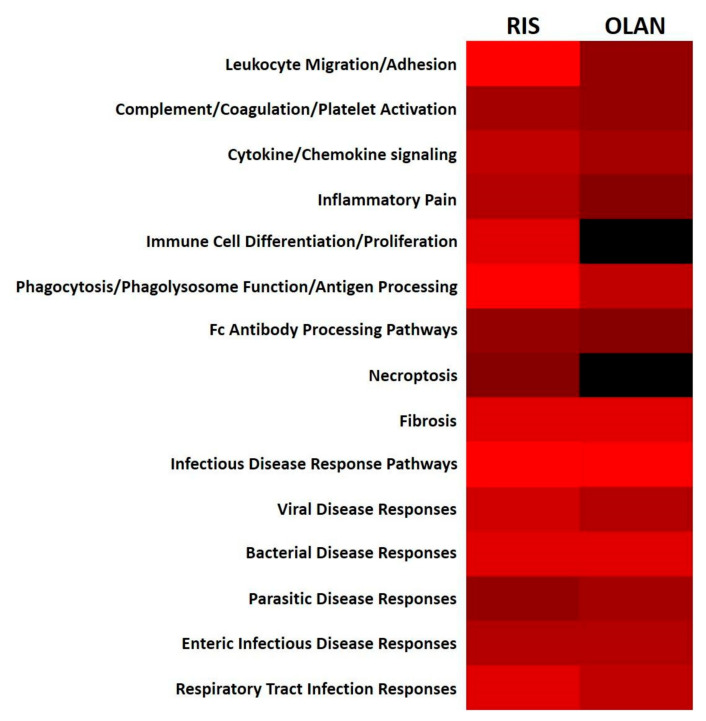
Dysregulation of immune function pathways during risperidone and olanzapine treatment. DE proteins measured in this study are integral parts of several immune function pathways in the KEGG database. The number of proteins altered in RIS- or OLAN-treated mice (relative to VEH-treated controls) in each pathway group were tabulated. The intensity of red (λ = 100–255 nm) reflects the number of proteins per pathway significantly different from VEH-treated mice during RIS treatment (left panel) and OLAN treatment (right panel). Absence of any altered proteins in a particular pathway is depicted in black (λ = 0 nm).

Insulin resistance is an inflammatory condition [38,39,40], and disruption of the liver’s metabolic system activates immune and inflammatory pathways that can further exacerbate metabolic perturbations that feed forward to NAFLD (Figure 4). These perturbations include tissue damage, steatohepatitis, and fibrosis [41,42]. Hepatocyte; hepatic stellate cell; sinusoidal endothelial cell; and Kupffer cell stress responses to lipotoxicity, metabolic dysregulation, and oxidative stress can all increase inflammatory cytokine release, innate intracellular immune reactivity, and immune cell recruitment and activation [43,44,45,46].

Both RIS and OLAN increased predicted activation of inflammatory pathways (Figure 4). Various intracellular mechanisms predicted to be activated by the dysregulated proteins contributed to this activation, including increased Nuclear Factor kappa-B (NF-κB) expression, increased expression of receptors for advanced glycation end products (RAGE) and pattern-recognition receptors, increased intracellular calcium, increased stress fiber formation, increased senescence, and increased prostaglandin formation. RIS-mediated upregulation of PRKCA, CTSS, GNAS, ROCK2, KLKB1, ATIC, IL1RAP, DGKZ, HSPB1, and RNF31, and OLAN-mediated upregulation of GLB1, SGPL1, CD68, and FMOD predicted increases in inflammatory cytokine production. Some of the dysregulated proteins also had predicted anti-inflammatory functions, which may represent local attempts to restore tissue homeostasis. These DE proteins were fewer in number, and included upregulated GSTA3, and downregulated CARD9, LAP3, and PSME1 by RIS, and upregulated G6PD and INPP5D, and downregulated PIP4P2 and ELAVL1 by OLAN.

Pathways that impact immune cell activation and migration were also predicted to be dysregulated by RIS and OLAN, including functions such as antigen processing, phagocytosis, Ras and MAP kinase signaling, and Nod signaling. RIS-induced upregulation of AFDN, LAMP1, PRKCA, and IL1RAP, and OLAN-induced upregulation of CFL1 and downregulation of CNTN1 and GNAI1 were predicted to increase immune cell activation and migration. Some of the dysregulated proteins also countered these predicted immune cell activations, such as RIS-induced downregulation of MSN and SNRPB, and OLAN-induced upregulation of INPP5D.

Some predicted intracellular functions serving as innate immune responses were also changed. These included predicted responses to numerous infectious diseases, necroptosis, antigen processing, immunogenic phospholipids, and protein misfolding. RIS-mediated downregulation of HYOU1, RPN2, GANAB, DDOST, ATIC, and AOX3, and upregulated RNF31, and OLAN-mediated upregulation of G6PD and NUP98, and downregulation of SEPT2, ACE, CYCS, and NCL were predicted to affect these functions.

A few of the dysregulated proteins were also predicted to affect changes in platelet and complement activation, potentially affecting coagulation and thrombotic conditions [47] as well as innate immune responses. RIS-mediated upregulation of KLKB1 and MBL1 (also increased by OLAN), and OLAN-mediated downregulation of GNAI1 were predicted to impact the coagulation cascade, platelet activation, and activation of the complement cascade.

### 2.7. Antipsychotic Treatment Affected Pathways Regulated by the Autonomic Nervous System: A Unifying Mechanism

The autonomic nervous system (ANS) exerts control over multiple physiological pathways in the liver, including those relating to insulin resistance and NAFLD [48]. Furthermore, AA medications have been implicated in regulating sympathetic nervous system (SNS) tone, and AA effects on heart and bone appear to be due at least in part to enhanced SNS activity [21,49,50,51]. In addition to pathways relating to insulin resistance, energy metabolism, fibrosis, and regeneration/repair described above, AA treatment altered the expression of proteins in other pathways known to be regulated by the ANS, including those relating to biliary physiology [48,52].

RIS and OLAN each had multiple DE proteins predicted to interfere with bile synthesis, secretion, and ductal motility. RIS-mediated DE of GNAS, BLVRB, AFDN, VAPA, LRP1, and HDS3B7 were all predicted to decrease proper processing and function of bile. With OLAN treatment, bile dysfunction was predicted due to the dysregulation of GNAI1, NPC2, VAPA, GLB1, AQP1, ATP2B4, TNNC2, and P2RX4.

**Figure 5:**
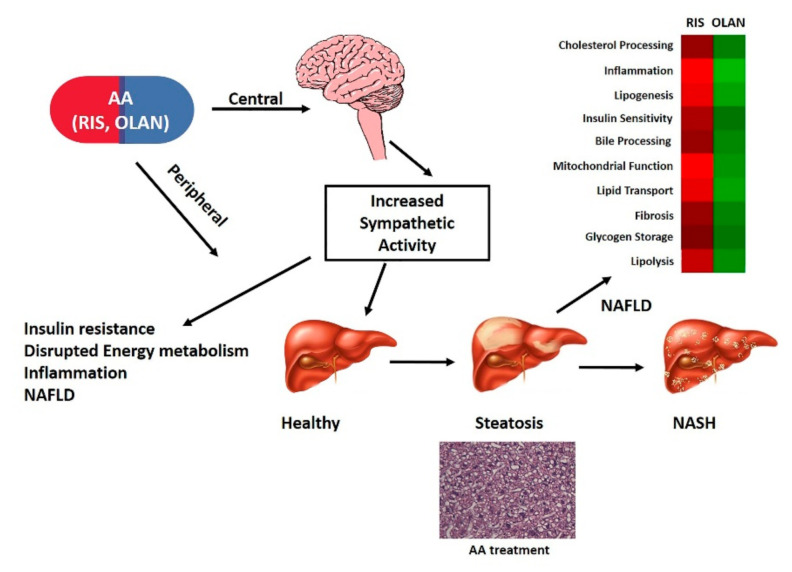
Model of antipsychotic regulation of NAFLD via sympathetic nervous system (SNS), metabolic, inflammatory, and endocrine factors. We propose that antipsychotic (AA) associated increase in sympathetic activity impacts the liver by causing alterations in the hepatic proteome and associated pathways leading to hepatic steatosis and NAFLD. A hematoxylin and eosin stained liver from OLAN-treated mouse at 20× magnification and pathway analysis heat maps (red-shading—RIS; green-shading—OLAN) are shown in support of this model.

Given the overarching regulatory role of the ANS in whole-body and hepatic functions linked to antipsychotic side effect profiles, AA-associated enhanced SNS tone is a powerful unifying regulatory system for drug associated metabolic side effects including NAFLD (Figure 5).

## 3. Discussion

Patients prescribed AA medications carry significant metabolic and cardiovascular risk burden, across the lifespan and regardless of diagnosis. Although the cardiovascular/metabolic risk varies in severity across the drug class, given the large patient population (on- and off-label prescribing) and added cardiovascular risk associated with severe mental illness, the overall public health risk and impact is high. Incidence of NAFLD is comorbid with obesity, insulin resistance/metabolic syndrome, as well as with severe mental illness [3,10]. NAFLD is a distinct risk factor for cardiovascular disease, and AA medications have been reported to increase NAFLD, clinically. Despite the extensive clinical literature characterizing the adverse endocrine and metabolic side effects of AA medications, the underlying molecular/pharmacologic mechanisms remain elusive. In order to specifically address the role of AA medications in the pathogenesis of NAFLD, we chose a proteomic approach, given the complexity of AA pharmacology as well as the complexity of cardiovascular disease and its underlying risk factors. This approach has been employed by others to evaluate the multifactorial etiology of NAFLD progression spanning multi-organ, multi-system effects as well as comorbidities and sex differences associated with NAFLD [53].

To our knowledge, this is the first report that examines the impact of moderate duration, clinically relevant dosing of AA medications on the liver proteome. AA medications display complex pharmacology, potently antagonizing diverse G protein-coupled receptors and eliciting complex phenotypes clinically; thus, a pathways analysis approach is needed to help tease out canonical and tissue-specific effects associated with these medications. Use of a preclinical model to evaluate potential mechanisms correlated with clinical phenotypes is a powerful approach as it (a) removes confounding effects of the disease on hepatic function/metabolic disease; (b) allows control of extrinsic factors such as nutrition, which is a known contributor to NAFLD (high fat diet); and (c) allows evaluation of complex, interconnected pathways involved in the chronic disease phenotypes induced by antipsychotic treatment. Our intentional use of C57BL/6 J mice fed a low-fat (chow) diet was to minimize the possibility that any AA-induced changes could be attributed to confounding effects of diet on dyslipidemia and metabolic disease.

Taking a proteomic approach allows for a broad, wide-ranging analysis in order to begin proposing mechanisms of AA-induced NAFLD. It should be noted that the DE proteins measured are almost certainly an underestimation of those affected, because this approach does not account for posttranslational modifications. Many of the pathways highlighted are regulated and mediated by phosphorylation events, and thus changes in fine-scale rheostasis of a pathway would not be detected by this approach. However, overt changes in protein level in AA-treated animals relative to VEH-treated controls are almost certainly biologically meaningful, particularly when the proteins themselves initiate downstream cascades. One such protein that is not activated by phosphorylation, mannose-binding lectin (MBL1), was significantly overexpressed during both RIS and OLAN treatment. The downstream impact of MBL1 interactions include activation of numerous phosphorylation-dependent signaling cascades (e.g., NF-κB signaling), some of which were not seen as changes in total proteins but whose impact could be seen phenotypically in the current report (Figure 1), in our prior reports demonstrating global immune dysregulation during AA treatment [54,55], and in several small-scale studies of patients taking AA medications [56]. Similarly, significantly depressed levels of cytochrome oxidase subunit VI B (COX6B) during both RIS and OLAN treatment would lead to profound changes in oxidative phosphorylation and metabolic rate that may not be reflected as level changes of downstream proteins because differentially phosphorylated states are not detected. However, metabolic changes in AA-treated patients and our previous report phenotypically demonstrate mitochondrial dysfunction that is highly consistent with reduced oxidative phosphorylation [21]. Finally, DE by both RIS and OLAN of proteins whose function depends on their phosphorylation state are highly likely to be relevant in the absence of posttranslational modification detection when the predicted phenotype is consistent with known AA-induced side effects. One such protein is smoothelin (SMTN1), which mediates contractility of smooth muscle such as that found in the vasculature. Significant changes in SMTN1 would be predictive of altered vascular contractility, leading to changes in blood pressure, and acute-onset changes in blood pressure are known side effects of AA, including both OLAN and RIS. All of these proposed mechanisms required further analysis to determine the impact of posttranslational modifications; however, the goal of this report was to describe an overarching framework with which to generate and evaluate specific mechanistic hypotheses addressing the development of AA-induced NAFLD.

It is well documented in the clinical literature that patients treated with AA medications often have increased adiposity, insulin resistance, dyslipidemia, and other related endocrine and metabolic side effects (see reviews [15,57,58]). Medication effects vary in severity across the AA drug class, with OLAN and clozapine noted for significant metabolic liability while RIS has intermediate metabolic liability [20]. These effects are observed across the lifespan in patients with severe mental illness, including schizophrenia, as well as in patients who are treated for other reasons [3,57,59]. This results in significant diminution in quality of life, shortening of lifespan, and increased burden on the healthcare system. Here, we focused on AA effects on the liver as insulin resistance is a pivotal factor underlying NAFLD progression [3,10]. The experimental design we employed culminated in significant hepatic steatosis, hepatocyte ballooning, formation of Mallory–Denk bodies, and development of inflammatory foci consistent with NAFLD (Figure 1). Coincident with this liver histopathology, we quantified significant changes in the liver proteomes of these mice, consistent with NAFLD. Low-dose, moderate duration of AA treatment resulted in a large number of DE proteins in RIS and OLAN mice. In this report, we chose to highlight DE profiles of proteins involved in pathways that are reported to be involved in NAFLD pathophysiology, including those regulating insulin sensitivity, energy metabolism, inflammatory/immune function, biliary function, fibrosis, and carcinogenesis.

It is not surprising, given the well-documented metabolic side effects observed clinically, that canonical pathways relating to energy homeostasis (including glucose, lipid, and amino acid metabolism and regulation of insulin sensitivity) were significantly altered in the livers of mice treated with RIS or OLAN. What is somewhat surprising, however, is the sheer number of DE proteins and canonical metabolic pathways perturbed by AA treatment. The magnitude of AA-associated effects we report here, observed in the absence of other extrinsic effects (such as high-fat feeding) or intrinsic effects of severe mental illness, provides mechanistic context to the well-documented, rapid, and profound metabolic dysregulation observed both clinically and preclinically.

There is growing evidence that schizophrenia has an inflammatory component [59], and previous clinical reports have suggested altered cytokine profiles in patients taking AAs [60,61,62]. This has led to the hypothesis that dampening of inflammatory responses may contribute to the efficacy of AA medications for psychosis. A unified mechanistic understanding of immune dysregulation has been difficult to decipher from these clinical reports because they are largely confounded by examining patients with psychiatric diagnoses and thus atypical inflammatory backgrounds. We previously reported global immune dysregulation with and without challenge, as measured by circulating cytokines and antibody levels using our preclinical murine model [54,55]. This current study bolsters these findings by identifying tissue-specific inflammatory dysfunction that could lead to NAFLD in the context of histopathologic findings consistent with its development.

Drug effects on hepatic function are likely to be both indirect (CNS mediated) and direct (tissue/cell mediated), as AA target receptors are expressed in the periphery as well as in the CNS. Direct and indirect regulation of AA-associated effects have been reported using preclinical models evaluating AA-associated whole-body and hepatic insulin resistance [25,26,27] and AA-associated effects on bone biology [63,64]. In the case of the liver, AA receptor targets are expressed on hepatocytes, and our proteomic analysis shows alterations in expression of proteins associated with GCPR signaling, consistent with direct effects of AA on the liver. This was most noticeable in the direct upregulation of the G_s_ G-protein (GNAS) by RIS, and the direct downregulation of the G_i_ G-protein (GNAI1) by OLAN, both of which were featured in numerous KEGG pathway maps. Globally impaired metabolic pathways, especially deficient production of amino acids, nucleic acids, and ATP, also limit the mitigation of NAFLD by inhibiting cell replication and repair mechanisms [65].

The liver is innervated by both afferent and efferent autonomic nerves, and myriad hepatic functions are regulated by the autonomic nervous system [48,66,67]. ANS-mediated hepatic functions include regulation of glucose and lipid metabolism, nutrient sensing, ion sensing and osmotic pressure, drug metabolism (CYP enzymes), blood flow, the biliary system, circadian rhythm, and hepatic regeneration and repair. Furthermore, the ANS has been implicated in the pathogenesis and progression of NAFLD [7]. Autonomic dysfunction is a hallmark of psychiatric as well as metabolic disease [68,69,70,71], and some side effects associated with AA therapy are consistent with medication-associated increase in sympathetic tone [50,58]. Some AA drugs have been reported to act via SNS mechanisms to cause adverse effects on glycemia [72], cardiac function [21,49,73], and bone biology [51,63]. In this report, both RIS and OLAN cause robust histological and proteomic changes in the liver that are consistent with NAFLD and dysregulation of myriad pathways associated with NAFLD and known to be regulated by the ANS. Thus, AA-associated disruption in ANS function is likely to be a pivotal overarching cause of AA-associated NAFLD (Figure 5).

In summary, low dose, moderate duration AA treatment leads to hepatic steatosis and fibrosis, preclinically, and is associated with hepatic proteomic signatures consistent with NAFLD. Patients with NAFLD have enhanced sympathetic nervous system activity (as do patients with schizophrenia and patients with diabetes). AA medications also are associated with increased sympathetic tone that has been implicated in AA-associated side effects of tissues including bone and heart, and effects on the liver proteome are consistent with increased sympathetic tone. Given the high prevalence of metabolic disease, including NAFLD, in psychiatric patients and the high rate of off-label prescribing of AA medications to non-psychotic patients including children and older adults, the likely impact of AA prescribing on population incidence of NAFLD is significant. Furthermore, as there is currently no US Food and Drug Administration (FDA)-approved therapeutic for NAFLD, increasing our understanding of the organ-specific as well as the multi-system mechanisms underlying disease pathology as well as elucidating pharmacological mechanisms associated with AA-induced NAFLD can aid in identifying novel therapeutic targets for this rapidly growing, global patient population.

## 4. Materials and Methods

### 4.1. Animals and Experimental Design

This study utilized 8-week-old male C57BL/6 J mice (The Jackson Laboratory, Bar Harbor, ME, USA) fed a standard chow diet for the duration of the study (28 days). We deliberately chose to maintain these animals on a standard, low-fat mouse chow diet in order to test effects of AA treatment per se, as high fat diets are known to induce insulin resistance and metabolic disease in this model. Animals were randomly assigned to 1 of 3 treatments: (a) vehicle (VEH) control, (b) risperidone (RIS), or (c) olanzapine (OLAN). At the culmination of the study, animals were euthanized by intraperitoneal (IP) injection of 2.5% avertin (in PBS) followed by cardiac perfusion. Livers were extracted following perfusion and were either snap frozen in liquid nitrogen for RNA and protein analysis or fixed in 4% paraformaldehyde and paraffin embedded. All animal experiments were carried out in compliance with the federal animal welfare laws and policies using protocol # 052918-003 (approved for operation from June 2018 until June 2021) approved by the Institutional Animal Care and Use Committee (IACUC) at the University of New England, Biddeford, ME, USA, following the National Institutes of Health guide for the care and use of laboratory animals [74].

### 4.2. Drug Formulation and Dosing Strategy

The dosing strategy employed was designed to achieve peak plasma drug concentrations that fall within the range of plasma drug exposures observed clinically in patients [24,75]. Briefly, animals were administered drug or vehicle via once daily oral (PO) gavage for 4 weeks (28 days). Doses included VEH (0.1 % acetic acid), RIS (Sigma-Aldrich, St. Louis, MO, USA; 1.0 mg/kg), or OLAN (Sigma-Aldrich, St. Louis, MO, USA; 5 mg/kg). Our dosing strategy was based on pharmacokinetic studies previously conducted in our laboratory using male and female mice [54,61].

### 4.3. Histopathology

Sections of each liver lobe were collected during necropsy and post-fixed in 4% paraformaldehyde overnight. Tissues were embedded in paraffin and 5 mm sections were taken for staining using hematoxylin and eosin (H&E) in order to qualitatively assess changes in pathology. Sections from each lobe were examined via brightfield microscopy at 20× magnification using a Keyence BZ-X710 inverted wide field digital microscope. Images were collected using BZ-X analyzer software. Histopathologic lesions were evaluated using a modification of the scoring criteria of Jensen et al. [76]. Specific modifications included (1) evaluation of tissues for Mallory–Denk bodies (present = 1; absent = 0), (2) scoring of “steatosis” parameters as microsteatosis, and (3) evaluation of macrosteatosis (i.e., intercellular steatosis) (lipid < 5% fieldview = 0; lipid 5%–33% fieldview = 1; lipid 33%–66% of fieldview = 2; lipid > 66% of fieldview = 3). Lesions scores were averaged across lobes for each animal and reported as means per treatment group.

### 4.4. Proteomic Methodology

Livers from VEH-, RIS-, and OLAN-treated mice were homogenized in HEPES-Triton-Sodium-Glycerol (HTNG) lysis buffer (20 mM hydroxyethyl-piperazineethanesulfonic acid (HEPES), 150 mM NaCl, 1.5 mM MgCl2, 10% glycerol, 1% Triton-X 100, 1 mM ethylenediamine tretraacetic acid (EDTA), protease-inhibitor cocktail (Calbiochem)). Protein concentration of the supernatant was determined by Bicinchoninic acid assay (BCA, Pierce). A total of 40 μg of protein was used from each sample. Tryptic digests of protein samples were performed using the ProteoExtract digestion kit (Calbiochem). Tryptic peptides were then separated on an Ultimate RSLC system 3000 (ThermoFisher/Dionex) nanoscale liquid chromatograph and infused onto a 5600 TripleTOF mass spectrometer (Sciex). Sequential window acquisition of all theoretical spectra (SWATH) was used to profile all proteins in each sample using a data-independent acquisition method. A mouse-specific ion library comprising 4091 proteins was constructed using ProteinPilot software (Sciex). For identification of peptides, we retrieved multiple fragment ion chromatograms from the spectral library for each peptide of interest. These spectra were compared with the extracted fragment ion traces for the corresponding isolation window to identify the transitions that best identify the target peptide. SWATH analysis was performed using PeakView software, and MarkerView software was utilized for principal component analysis and *t*-test comparisons [77]. Detailed proteomic analysis methods are available at PeptideAtlas (identifier: PASS01349). The proteomic data were analyzed in the following pairwise comparisons: liver RIS to liver VEH and liver OLAN to liver VEH. An adjusted *p*-value was calculated for each protein in each pairwise dataset using the False Data Recovery (FDR) method. Two significant protein lists were then made for each pairwise dataset using the raw and adjusted *p*-values with a threshold of 0.05. Proteins associated or phenotypes showing altered expression during AA treatment relative to VEH-treated controls were binned and tabulated for this study.

### 4.5. Proteomic Analysis

The proteomic data were analyzed comparing RIS or OLAN to vehicle control after log transformation to achieve normality. A differentially expressed (DE) protein list with a rounded *p*-value threshold of 0.05 was established for pathway analysis. Pathway analysis was performed using the Kyoto Encyclopedia of Genes and Genomes (KEGG) database, releases 95.0, 95.1, and 95.2 (https://www.genome.jp/kegg) [78,79,80]. DE proteins were queried on the KEGG, and all pathway maps and corresponding object identifier numbers attributed to each protein were recorded. DE proteins without KEGG pathway maps were excluded from subsequent evaluations. The role of each DE protein on each KEGG pathway map was determined on the basis of the relationships with other proteins and molecules with which it interacted, according to the KEGG notations key (https://www.genome.jp/kegg/document/help_pathway.html). Changes in predicted physiological function noted in each KEGG pathway map, upstream and/or downstream in the signaling pathways relative to the DE protein, and the effects on those predicted functions caused by the upregulation or downregulation of the DE proteins were recorded. The specific predicted functional changes caused by the change in expression of each DE protein were recorded separately for each combination DE protein/KEGG pathway map. Subsequent to all DE proteins being analyzed, we tabulated the frequency of each predicted physiological function’s increase (“up”) or decrease (“down”) for each drug. For the evaluation of larger systemic changes, we sorted the predicted physiological functions into classifications (categories) on the basis of similar biological functions (e.g., metabolic functions).

### 4.6. RNA Analysis

Liver tissue was homogenized in liquid nitrogen, total RNA was isolated by TriReagent/chloroform extraction, and RNA quality was assessed by Nanodrop (Thermo Fisher Scientific, Waltham, MA, USA). Total RNA was then analyzed using RT^2^ Profiler PCR Array (Mouse PPAR Targets #330231; Qiagen).

### 4.7. Statistical Analysis

All data are expressed as the mean ± standard error of the mean (SEM), unless otherwise specified. All statistical analysis was performed using Student’s *t*-test or ANOVA with post hoc analyses, as indicated unless specified. All statistical analysis was performed using Prism 8 statistical software (GraphPad Software, Inc., La Jolla, CA, USA).

### 4.8. Data Statement

The proteomic datasets analyzed for this study can be found in the PeptideAtlas (identifier: PASS01349) http://www.peptideatlas.org/PASS/PASS01349. The raw data supporting the conclusions of this manuscript will be made available by the authors, without undue reservation, to any qualified researcher.

## Figures and Tables

**Table 1:** Selected results from the KEGG function categories for differentially expressed hepatic proteins from mice treated with risperidone.

	Predicted Dysregulated Functions with RIS	Up	Down	Associated Proteins
Predicted metabolic effects	Energy production (all ATP-generating pathways combined)	41	62	BCAT2, CS, AGXT, ABAT, NDUFA8, NDUFB8, NDUFS1, COX6B1, CYB5R3, ALDH7A1, TALDO1, ACAA2, ACLY, PRKCA, PDGFRB, GNAS, PGAM2, ENO3, LRP1, ALR1, AOX3, ADIPOQ, HIBADH, COMT, MTHFD1, ECI2, NUDT7, DGKZ, EPHA2, ITGA3, SLC27A1, BLVRB, SEC22B, SMARCC1, ASGR1, BTD
	Amino acid production	32	37	BCAT2, CS, ADIPOQ, LAP3, PGAM2, TALDO1, ENO3, ABAT, ACLY, PCBD2, ALR1, DGKZ, GNMT, AGXT, ALDH7A1, PRKCA, GNAS, FTCD, AMDHD1, ATIC, MTHFD1, EPHA2, PDGFRB, ITGA3, AOX3, COMT, ACAA2, HIBADH, TCN2
	Glycolysis	36	7	ABAT, ACLY, ALDH7A1, AOX3, COX6B1, CS, EPHA2, GNAS, ITGA3, LAP3, NDUFS1, PDGFRB, PGAM2, PRKCA, TALDO1, ALR1, ENO3,
	Lipolysis	27	9	ABAT, ACAA2, ACLY, ADIPOQ, ALDH7A1, BLVRB, COX6B1, CS, ECI2, GNAS, MECR, PDGFRB, PRKCA, SLC27A1, AOX3, NUDT7
	Lipogenesis	24	2	ABAT, ACAA2, ADIPOQ, ALDH7A1, ALR1, AOX3, DGKZ, GNAS, MECR, PRKCA, SLC27A1, TCN2, SEC22B
	TCA/OxPhos/ETC	16	9	ADIPOQ, BCAT2, NDUFA8, NDUFB8, PGAM2, ALDH7A1, ALR1, COX6B1, ENO3, NDUFS1
	Nucleic acid synthesis	7	13	EPHA2, GNAS, ITGA3, PDGFRB, PRKCA, SMARCC1, TCN2, ALR1, AMDHD1, ATIC, FTCD, MTHFD1, NUDT7, TALDO1
	Lysosomal function	10	3	ADIPOQ, CTSS, GSTA3, HGS, LAMP1, RAB11FIP5, SUMF1, RPN2, SEC22B, VAPA
	Mitochondrial biogenesis	11	0	ADIPOQ, GNAS, PDGFRB, PRKCA, SLC27A1, SMARCC1
Predicted mitogenic effects	Migration/motility	55	21	DGKZ, ENAH, EPHA2, GNAS, HSPB1, IL1RAP, ITGA3, MSN, NDUFA8, PDGFRB, PRKCA, ROCK2, ADIPOQ, AFDN, ASGR1, ELAVL1, INPP5D, MYH9, NDUFS1
	Cell survival	49	6	CRYAB, DGKZ, EPHA2, GNAS, GSTA3, HSPB1, IL1RAP, ITGA3, NDUFA8, PDGFRB, PRKCA, RNF31, ADIPOQ, ASGR1, ELAVL1, INPP5D, NDUFS1
	Cell proliferation	46	6	DGKZ, EPHA2, GNAS, HSPB1, IL1RAP, ITGA3, NDUFA8, NDUFB8, PDGFRB, PRKCA, ADIPOQ, ASGR1, ELAVL1, INPP5D, NDUFS1
	Protein production	13	37	EIF5, EPHA2, FAU, GNAS, HNRNPC, HSPB1, ITGA3, PDGFRB, PRKCA, TRA2B, AARS, ABAT, AGXT, ALDH7A1, ALR1, AMDHD1, ATIC, CRYAB, DDOST, EIF5B, FTCD, GANAB, HGS, HYOU1, NOP56, PABPC1, PUF60, RAB11FIP5, REX02, RPN2, RPS3A1, SEC22B, SNRPB, SNRPG, TALDO1, TARS
	Cancer	25	7	DGKZ, GNAS, GSTA3, ITGA3, KLK3, PDGFRB, PRKCA, ROCK2, EPHA2, HPGD, SMARCC1
Predicted tissue-damaging effects	Oxidation/ROS generation	34	10	ABAT, AGXT, ALDH7A1, ALR1, AOX3, BLVRB, COMT, COX6B1, DDOST, EPHA2, GANAB, GNAS, HGS, YOU1, ITGA3, NDUFA8, NDUFB8, PDGFRB, PRKCA, RAB11FIP5, ROCK2, RPN2, ADIPOQ, COX6B1, GSTA3, LAP3, NDUFS1, SMARCC1,
	Disrupted elimination of toxins/xenobiotics	8	7	GSTA3, LAP3, ALR1, AOX3, COX6B1, NUDT7, TALDO1
	Protein misfolding	9	1	DDOST, GANAB, LAP3, RPN2, VCP

**Table 2:** Selected results from the KEGG function categories for differentially expressed hepatic proteins from mice treated with olanzapine.

	Predicted Dysregulated Functions of OLAN	Up	Down	Associated Proteins
Predicted metabolic functions	Energy production (all ATP-generating pathways combined)	7	27	G6PD, GLB1, NPC2, RPL34, AK2, AMY2B, ATP2B4, COX6B1, ENO3, GNAI, PNP, TNNC2, VAPA
	Nucleic acid synthesis	7	15	G6PD, MTHFD1L, AK2, ATIC, ENO3, MTAP, PNP
	Amino acid production	7	10	G6PD, GLB1, MTHFD1L, ATIC, ENO3, MTAP, PNP
	Glycolysis	6	10	ATP2B4, G6PD, GLB1, P2RX4, AK2, AMY2B, ENO3
	Lipogenesis	12	5	AGPAT3, G6PD, GNAI, HMGA2, HNRNPD, NPC2, ATP2B4, ENO3, VAPA
	Lipolysis	5	10	AGPAT3, GNAI1, NPC2, AMY2B, COX6B1, CYCS, DECR2, ENO3, VAPA
	TCA/OxPhos/ETC	4	9	G6PD, GLB1, RPL34, COX6B1, CYCS, ENO3, PNP
	Glycogen production	2	9	ENO3, GNAI, ATP2B4, P2RX4
Predicted mitogenic effects	Cell survival	21	14	AGPAT3, ATP2B4, CYCS, FMOD, GNAI1, P2RX4, SGPL1, GLB1, GNAI1, INPP5D, LUM, PIP4P2, PNP, TOP2B
	Cell proliferation	8	15	AGPAT3, FMOD, GLB1, GNAI1, SGPL1, ATP2B4, CTC1, ELAVL1, INPP5D, P2RX4, PIP4P2, SGPL1, TOP2B
	Cancer	13	5	CYCS, G6PD, GNAI, HMGA2, PLOD3, LUM, TOP2B, TRIM32
	Migration/motility	6	9	AGPAT3, CFL1, GLB1, SGPL1, ATP2B4, GNAI1, INPP5D, PIP4P2
	Protein production	7	6	FAU, HNRNPC, NUP98, RPL34, RPL8, RPS15, RPS4Y1, ATP2B4, GNAI, RNPS1, TOP2B
Predicted immune effects	Inflammation/cytokines	23	9	ACE, AMY2B, ATIC, CD68, CFL1, CNTN1, CYCS, G6PD, GLB1, GNAI1, MBL1, SGPL1, ELAVL1, INPP5D, MTHFD1L
	Immune activation	10	13	CD68, CFL1, DYNCH1H1, FMOD, G6PD, GLB1, GNAI, MBL1, AK2, CNTN1, CYCS, ELAVL1, INPP5D, PIP4P2, PNP
	Cell microbe response	14	7	ACE, CFL1, CYCS, GNAI1, NCL, NUP98, SEPT2
Predicted GI/nutrient handling	GI function/nutrient absorption	5	17	GLB1, GNAI1, NPC2, AMY2B, AQP1, ATP2B4, TNNC2, VAPA
	Bile processing	5	10	GLB1, GNAI1, NPC2, AQP1, ATP2B4, P2RX4, TNNC2, VAPA
	Cholesterol processing	5	5	AMY2B, HNRNPD, NPC2, AQP1, VAPA
Predicted tissue damaging effects	Oxidation/ROS generation	17	3	AMY2B, CNTN1, COX6B1, CYCS, GLB1, GNAi1, PLOD3, PNP, G6PD
	Fibrosis	9	1	COX6B1, FMOD, GLB1, GNAI, PLOD3, NPC2
Predicted intracellular functions	Cytoskeletal dynamics/intracellular transport	13	5	CFL1, CMT4F, DYNCH1H1, GNAI1, RAB31, SRP19, VPS13C, PIP4P2, SEPT2, VAPA
	Export of intracellular calcium ions	8	2	ATP2B4, P2RX4, GNAI1
Predicted Blood physiology	Blood pressure	7	7	ACE, DYNCH1H1, GNAI1, AQP1, ATP2B4

**Table 3:** Significantly overrepresented proteins differentially expressed in livers from animals treated with risperidone or olanzapine.

**Risperidone**					
**Upregulated**	**Name**	**# KEGG Pathways**	**Downregulated**	**Name**	**# KEGG Pathways**
PRKCA	Protein kinase C alpha	53	ALDH7A1	Aldehyde dehydrogenase 7, member a1	14
GNAS	Guanine nucleotide-binding protein, alpha-stimulating	40	COX6B1	Cytochrome C oxidase subunit 6B1	11
PDGFRB	Platelet-derived growth factor receptor beta	18	AOX3	Aldehyde oxidase 3	9
ROCK2	Rho-associated coiled-coil-containing protein kinase 2	15	ENO3	Enolase 3	8
ITGA3	Integrin alpha 3	11	AGXT	Alanine-glyoxylate and serine-pyruvate aminotransferase	7
GSTA3	Glutathione-S-transferase A3	10	CS	Citrate synthase oxidoreductase core subunit S1	7
NDUFA8/ NDUFB8	Inner mitochondrial membrane complex 1 NADH:ubiquinone oxidoreductase subunit A8/B8	7/7	NDUFS1	NADH ubiquinone	7
PGAM2	Phosphoglycerate mutase 2	7	ABAT	4-Aminobutyrate aminotransferase	7
BCAT2	Branched-chain amino acid transaminase 2	7	ALR1	Aldo-keto reductase family 1 member B	6
AFDN	Afadin	6			
INPP5D	Inositol polyphosphate 5-phosphatase D	6			
ACAA2	Acetyl CoA acyltransferase 2	6			
ADIPOQ	Adiponectin, C1Q, and collagen domain-containing	6			
DGKZ	Diacylglycerol kinase-zeta	6			
EPHA2	EPH-ephrin receptor A2	5			
**Olanzapine**					
**Upregulated**	**Name**	**# KEGG Pathways**	**Downregulated**	**Name**	**# KEGG Pathways**
GLB1	Beta-galactosidase	12	GNAI1	G-protein subunit alpha inhibitory-1	37
G6PD	Glucose 6 phosphate dehydrogenase	10	CYCS	Somatic cytochrome C	26
ATP2B4	ATPase plasma membrane Ca+2 transporting 1	9	COX6B1	Cytochrome C oxidase subunit 6B1	11
INPP5D	Inositol polyphosphate 5-phosphatase D	6	ENO3	Enolase 3	8
AGPAT3	1-Acylglycerol-3-phosphate O-acyltransferase-3	5	ACE	Angiotensin I-converting enzyme	7
CFL1	Cofilin 1	5	PNP	Purine nucleoside phosphorylase	6
			AK2	Adenylate kinase 2	5
			ATIC	5-Aminoimidazole-4-carboxamide ribonucleotide formyltransferase/IMP cyclohydrolase	5
			AMY2B	Amylase alpha 2B	5

KEGG (Kyoto Encyclopedia of Genes and Genomes) pathway analysis of risperidone- and olanzapine-associated changes in liver protein expression compared to VEH controls. #—indicates “number”. “Up” and “Down” respectively indicate increased or decreased instances of each function on KEGG pathways as a result of changes in the associated protein expression (see Supplementary File S3 for detailed correlations). RIS *n* = 5; OLAN *n* = 4; VEH *n* = 4. Abbreviations: RIS, risperidone; OLAN, olanzapine; OxPhos, oxidative phosphorylation; TCA, tricarboxylic acid cycle; ETC, electron transport chain; GI, gastrointestinal; ROS, reactive oxygen species.

## Supplementary Materials

The following are available online at https://www.mdpi.com/1422-0067/21/24/9362/s1. Five supplementary data files are provided in support of this manuscript.
