# Supplementary material for: Understanding Mechanisms Underlying Non-Alcoholic Fatty Liver Disease (NAFLD) in Mental Illness: Risperidone and Olanzapine Alter the Hepatic Proteomic Signature in Mice
Source: Int J Mol Sci. 2020 Dec 8;21(24):9362. doi: 10.3390/ijms21249362 (PMC7763698; doi:10.3390/ijms21249362)
Supplement: Supplementary file 1 [file ijms-21-09362-s001.zip › ijms-1004946-supplementary/Revised manuscript and supplemental data file/S1_Title_Legend.docx]

Supplemental File 1: “1_MassSpec_Proteome_Data”

Title: *Mass Spectrometry Proteome Data for Risperidone and Olanzapine Compared to Vehicle*

Legend:

Raw and annotated data of proteins that were differentially detected in livers of mice treated with risperidone (RIS) or olanzapine (OLAN) relative to livers of respective vehicle-treated negative-control mice. Proteome data was obtained via LC-MS/MS from tryptic digests of homogenized, pooled livers of mice (RIS n = 5, OLAN n = 4, VEH n = 4), and statistical significance was determined by PCA and *t-*test comparisons using MarkerView software.
