# Supplementary material for: Understanding Mechanisms Underlying Non-Alcoholic Fatty Liver Disease (NAFLD) in Mental Illness: Risperidone and Olanzapine Alter the Hepatic Proteomic Signature in Mice
Source: Int J Mol Sci. 2020 Dec 8;21(24):9362. doi: 10.3390/ijms21249362 (PMC7763698; doi:10.3390/ijms21249362)
Supplement: Supplementary file 1 [file ijms-21-09362-s001.zip › ijms-1004946-supplementary/Revised manuscript and supplemental data file/S2_Title_Legend.docx]

Supplemental File 2: “2_Proteome_KEGG_data”

Title: *KEGG Pathways Associated with Significantly Changed Proteins in the Livers of Risperidone- and Olanzapine-Treated Mice*

Legend:

Proteins obtained from the mass spectrometry proteomics data for risperidone and olanzapine were sorted for statistical significance (p < 0.055), and compared individually against the KEGG (Kyoto Encyclopedia of Genes & Genomes, <https://www.genome.jp/kegg>) database. Proteins that had curated KEGG pathway maps were selected and listed together with all of the KEGG maps and the live URLs on their respective spreadsheets, and sorted for up- or downregulation relative to vehicle controls.
