# Supplementary material for: Understanding Mechanisms Underlying Non-Alcoholic Fatty Liver Disease (NAFLD) in Mental Illness: Risperidone and Olanzapine Alter the Hepatic Proteomic Signature in Mice
Source: Int J Mol Sci. 2020 Dec 8;21(24):9362. doi: 10.3390/ijms21249362 (PMC7763698; doi:10.3390/ijms21249362)
Supplement: Supplementary file 1 [file ijms-21-09362-s001.zip › ijms-1004946-supplementary/Revised manuscript and supplemental data file/S3_Title_Legend.docx]

Supplemental File 3: “3_RIS_OLAN_Pathway_analyses”

Title: *KEGG Pathway Analysis and Resulting Functional Effects of Liver Proteins Altered via Risperidone and Olanzapine Treatment of Mice*

Legend:

Proteins changed as a result of risperidone (RIS) or olanzapine (OLAN) treatment, for which KEGG (Kyoto Encyclopedia of Genes & Genomes, <https://www.genome.jp/kegg>) pathway maps existed were analyzed for the potential changes in pathway function as a result of their up- or downregulation. Functional changes were determined specifically by analysis of each KEGG pathway up- or downstream of the altered protein, including instances where the protein appeared multiple times on different sections of a map with different predicted functional effects. Predicted functional changes for each combination protein/map were recorded in the spreadsheet and tabulated, including redundant iterations of predicted functions that may have occurred on different KEGG pathway maps.
