# Supplementary material for: Understanding Mechanisms Underlying Non-Alcoholic Fatty Liver Disease (NAFLD) in Mental Illness: Risperidone and Olanzapine Alter the Hepatic Proteomic Signature in Mice
Source: Int J Mol Sci. 2020 Dec 8;21(24):9362. doi: 10.3390/ijms21249362 (PMC7763698; doi:10.3390/ijms21249362)
Supplement: Supplementary file 1 [file ijms-21-09362-s001.zip › ijms-1004946-supplementary/Revised manuscript and supplemental data file/S4_Title_Legend.docx]

Supplemental File 4: “4_Predicted_functions_counts_categories”

Title: *Changes in Predicted Functions and their Categories, Resulting from Up- or Down-Regulation of Liver Proteins As a Result of Risperidone or Olanzapine Treatment of Mice*

Legend:

The predicted physiological functional changes tabulated after KEGG (Kyoto Encyclopedia of Genes & Genomes, <https://www.genome.jp/kegg>) pathway analysis for each protein were grouped and categorized for quantification and determination of their trends. Shared predicted functional changes and groups were compared, based on total number of occurrences of a predicted function (up and down), most increased number of occurrences of increases in a predicted function (up) and most number of occurrences of decreases in a predicted function (down).
