# Supplementary material for: Understanding Mechanisms Underlying Non-Alcoholic Fatty Liver Disease (NAFLD) in Mental Illness: Risperidone and Olanzapine Alter the Hepatic Proteomic Signature in Mice
Source: Int J Mol Sci. 2020 Dec 8;21(24):9362. doi: 10.3390/ijms21249362 (PMC7763698; doi:10.3390/ijms21249362)
Supplement: Supplementary file 1 [file ijms-21-09362-s001.zip › ijms-1004946-supplementary/Revised manuscript and supplemental data file/S5 PPAR target genes.pptx]

## Slide 1
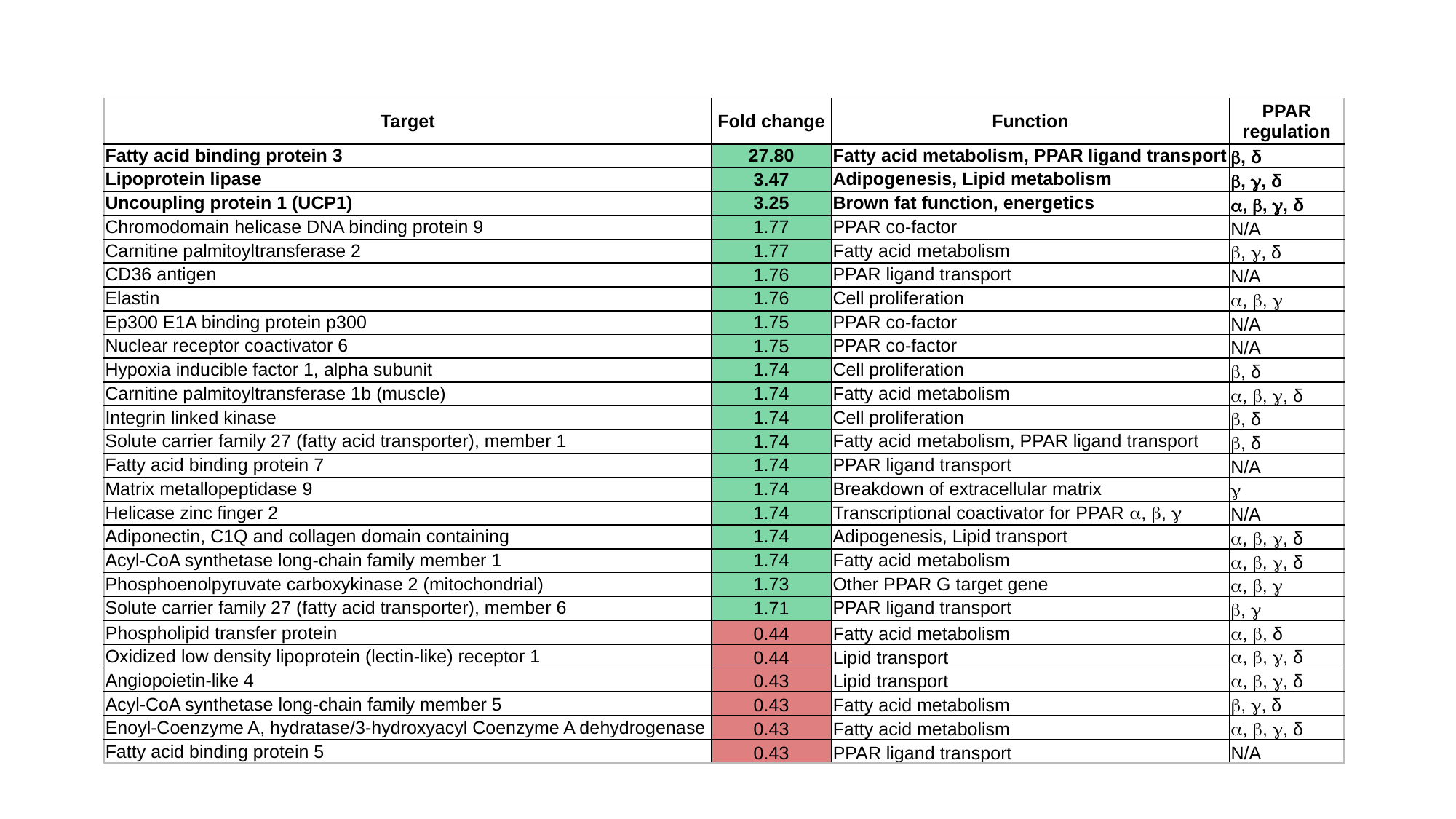

| Target | Fold change | Function | PPAR regulation |
| --- | --- | --- | --- |
| Fatty acid binding protein 3 | 27.80 | Fatty acid metabolism, PPAR ligand transport | β, δ |
| Lipoprotein lipase | 3.47 | Adipogenesis, Lipid metabolism | β, γ, δ |
| Uncoupling protein 1 (UCP1) | 3.25 | Brown fat function, energetics | α, β, γ, δ |
| Chromodomain helicase DNA binding protein 9 | 1.77 | PPAR co-factor | N/A |
| Carnitine palmitoyltransferase 2 | 1.77 | Fatty acid metabolism | β, γ, δ |
| CD36 antigen | 1.76 | PPAR ligand transport | N/A |
| Elastin | 1.76 | Cell proliferation | α, β, γ |
| Ep300 E1A binding protein p300 | 1.75 | PPAR co-factor | N/A |
| Nuclear receptor coactivator 6 | 1.75 | PPAR co-factor | N/A |
| Hypoxia inducible factor 1, alpha subunit | 1.74 | Cell proliferation | β, δ |
| Carnitine palmitoyltransferase 1b (muscle) | 1.74 | Fatty acid metabolism | α, β, γ, δ |
| Integrin linked kinase | 1.74 | Cell proliferation | β, δ |
| Solute carrier family 27 (fatty acid transporter), member 1 | 1.74 | Fatty acid metabolism, PPAR ligand transport | β, δ |
| Fatty acid binding protein 7 | 1.74 | PPAR ligand transport | N/A |
| Matrix metallopeptidase 9 | 1.74 | Breakdown of extracellular matrix | γ |
| Helicase zinc finger 2 | 1.74 | Transcriptional coactivator for PPAR α, β, γ | N/A |
| Adiponectin, C1Q and collagen domain containing | 1.74 | Adipogenesis, Lipid transport | α, β, γ, δ |
| Acyl-CoA synthetase long-chain family member 1 | 1.74 | Fatty acid metabolism | α, β, γ, δ |
| Phosphoenolpyruvate carboxykinase 2 (mitochondrial) | 1.73 | Other PPAR G target gene | α, β, γ |
| Solute carrier family 27 (fatty acid transporter), member 6 | 1.71 | PPAR ligand transport | β, γ |
| Phospholipid transfer protein | 0.44 | Fatty acid metabolism | α, β, δ |
| Oxidized low density lipoprotein (lectin-like) receptor 1 | 0.44 | Lipid transport | α, β, γ, δ |
| Angiopoietin-like 4 | 0.43 | Lipid transport | α, β, γ, δ |
| Acyl-CoA synthetase long-chain family member 5 | 0.43 | Fatty acid metabolism | β, γ, δ |
| Enoyl-Coenzyme A, hydratase/3-hydroxyacyl Coenzyme A dehydrogenase | 0.43 | Fatty acid metabolism | α, β, γ, δ |
| Fatty acid binding protein 5 | 0.43 | PPAR ligand transport | N/A |
