# Supplementary material for: Understanding Mechanisms Underlying Non-Alcoholic Fatty Liver Disease (NAFLD) in Mental Illness: Risperidone and Olanzapine Alter the Hepatic Proteomic Signature in Mice
Source: Int J Mol Sci. 2020 Dec 8;21(24):9362. doi: 10.3390/ijms21249362 (PMC7763698; doi:10.3390/ijms21249362)
Supplement: Supplementary file 1 [file ijms-21-09362-s001.zip › ijms-1004946-supplementary/Revised manuscript and supplemental data file/S5_Title_Legend.docx]

Supplemental File 5: “Supplementary figure 5 PPAR target genes”

Title: *RNA Expression of PPAR Targets in Livers of Mice Treated with Olanzapine versus Vehicle*

Legend:

Total RNA extracted from livers of mice treated with vehicle (VEH) or olanzapine (OLAN) were analyzed using RT² Profiler™ PCR Array (Mouse PPAR Targets; GeneGlobe Id - PAMM-149Z; Qiagen Catalog No. **–**330231). Data are illustrated as fold change vs. VEH; *P <0.05*. Green = up-regulated vs. VEH; Red = down-regulated vs. VEH.
